# A study on the environmental characteristics and social support for physical activity among adolescents: implications for public health nursing

**DOI:** 10.3389/fpubh.2026.1835941

**Published:** 2026-05-21

**Authors:** Lifen Guo, Xielin Zhou, Qiang Guo, LeLe Lin

**Affiliations:** 1 The Affiliated People’s Hospital of Ningbo University, Ningbo, Zhejiang, China; 2 Faculty of Sports Science, Ningbo University, Ningbo, Zhejiang, China

**Keywords:** adolescent health, high school student, parental support, peer support, physical activity

## Abstract

**Objective:**

To investigate the distribution patterns of physical activity levels, perceptions of physical activity and the social support environment among secondary school students; to examine the associations between students’ physical activity levels and factors at the individual, peer and parental levels; and to analyse the dynamic relationships between physical activity and the social support environment across different dimensions, such as gender, year group and school.

**Methods:**

Using convenience sampling—a non-probability sampling method—we surveyed 841 high school students using the Adolescent Physical Activity Questionnaire, the Physical Activity Perception Questionnaire, and the Adolescent Physical Activity Social Support Scale. Statistical analyses were conducted using SPSS Statistics 26.0 to construct models via stepwise and hierarchical regression.

**Results:**

(1) As many as 46.0% of high school students reported low levels of physical activity. The mean peer support score for boys (14.46 ± 4.18) was higher than that for girls (12.55 ± 3.89). No significant differences were found in peer support or parental support across grade levels (*p* > 0.05). (2) There was no significant association between parental support and physical activity among secondary school students (*p* > 0.05). With regard to gender, there was a significant positive association between peer support and both perceptions of physical activity and levels of physical activity among boys (*p* < 0.01), whereas among girls, only the positive association with peer support was significant (*p* < 0.01).

**Conclusion:**

(1) High school students receive relatively little support from peers and parents; among them, boys receive more peer support than girls. (2) Peer support and perceptions of physical activity are significantly and positively associated with physical activity among secondary school students; this association exhibits different characteristics across gender groups, but no significant differences were found across year groups.

## 1 Introduction

The 2020 World Health Organization (WHO) Guidelines on Physical Activity and Sedentary Behavior recommend that children and adolescents engage in at least 60 min of moderate-to-vigorous physical activity daily and reduce the amount of time spent sitting and using recreational screens (1). In the same year, the “Global Action Plan on Physical Activity 2018–2030” further emphasized that building a supportive social environment through multisectoral collaboration is a key strategy for increasing physical activity levels among the general population (2). At the same time, countries around the world are placing increasing emphasis on the physical health of adolescents. The U.S. “Physical Activity Guidelines (Second Edition)” note that adolescence is a critical period for individuals to develop lifelong exercise habits, and that support from families, schools, and peers plays an irreplaceable role in their participation in physical activity (3); China’s “Healthy China 2030” Outline also calls for strengthening physical education for adolescents and emphasizes the collaborative efforts of multiple stakeholders to promote their healthy development (4). Evidently, against the backdrop of international consensus, how to enhance adolescents’ physical activities [activities such as entertainment, games, sports, transportation, and physical exercises that individuals engage in within the social environment (5)] has emerged as a core issue of common concern in the field of health promotion for this group. However, with the widespread use of electronic smart devices and the heavy academic pressure they face, many adolescents worldwide suffer from a severe lack of physical exercise, a phenomenon that is particularly prominent among high school students (6). According to 2023 monitoring data from China’s National Health Commission, the overall prevalence of myopia among children and adolescents is as high as 52.7%, with the rate among high school students exceeding 80%, the highest in the world (7). The “China Cardiovascular Health and Disease Report 2022” also notes that less than 40% of children and adolescents meet physical activity guidelines, and health risks such as obesity, hypertension, and metabolic syndrome are becoming increasingly prominent (8). As can be seen, the low levels of physical activity among Chinese adolescents and their less-than-ideal health status represent a major public health challenge facing society today, and there is an urgent need to explore the underlying causes of this problem.

Currently, it is widely accepted in academic circles that adolescents’ physical activity is influenced by social, psychological, and environmental factors, among which social support is one of the key factors affecting their level of physical activity (9). Social support theory posits that social support, in various forms (such as emotional support, informational support, and instrumental support), can mitigate the negative effects of stress on individuals, thereby improving their overall health and quality of life (10). In physical activity settings, providing active support to individuals who are about to begin or are already engaged in physical exercise may effectively promote their participation in physical activity (11). Research indicates that there is a significant positive correlation between adolescents’ levels of physical activity and social support; parents and peers, as their primary sources of support, play a role through encouragement, joint participation, and practical assistance (12). However, from a theoretical perspective, existing research has not yet sufficiently explored the mechanisms underlying the role of social support. In the research on social support for adolescents’ physical activities [In the process of adolescents participating in physical activities, the various types of help provided by important others such as parents and peers to the individuals themselves regarding the social support for adolescents’ physical activities (13)], although there are already multiple measurement tools and classifications such as instrumental support, emotional support, and informational support, researchers mostly focus on the individual effect of a certain type of support provider, lacking the analysis of the interaction mechanism and path relationship of multi-source support in promoting physical activities among high school students (14). From a research perspective, existing studies have not thoroughly examined the mechanisms underlying the relationship between the type and source of support, making it difficult to fully elucidate the differential effects of different types of social support on high school students’ physical activity (15). From the perspective of theoretical integration, social support theory and the social ecological model have not yet been fully applied to research on the multi-level pathways for promoting physical activity among high school students (16). High school is a critical stage in adolescent development, during which students undergo rapid physical and psychological growth, and their behavioral patterns are influenced by both family and peers. However, the mechanism by which different types of support influence individuals’ perceptions of physical activity and, in turn, affect their actual participation has not yet been fully elucidated (17). Therefore, to overcome the limitations of previous studies, this research draws upon social support theory and the social ecological model. Data were collected using the Adolescent Physical Activity Questionnaire, the Physical Activity Perception Questionnaire, and the Adolescent Physical Activity Social Support Scale. SPSS was employed to analyze the relationship between high school students’ physical activity levels and various influencing factors, with the aim of understanding the distribution characteristics of their physical activity levels, perceptions of physical activity, and social support environments; It examines the influence of individual students, peers, and parents on high school students’ physical activity levels and analyzes the dynamic relationships between physical activity and the social support environment across different dimensions such as gender, grade level, and school. The aim is to provide quantitative evidence to clarify the effects of peer support, parental support, and perceptions of physical activity on high school students’ physical activity levels, as well as any gender differences, thereby deepening our understanding of the socio-ecological mechanisms underlying physical activity in this population.

## 2 Methods

### 2.1 Design

This study employed a non-probability convenience sampling method (which offers the advantages of high on-site organizational efficiency and a relatively high response rate). Four provincial key high schools (JSZX, XSZX, YZZX, and ZHZX) were selected as survey sites; these schools possess a certain degree of regional representativeness and reference value in terms of sports facilities, teaching staff quality, and students’ family socioeconomic status. The total enrollment at the four schools is approximately 1,700, 1,800, 1,500, and 1,300 students, respectively, for a combined total of about 6,300 students (the combined total of students in the 10th and 11th grades at these schools is approximately 3,800). The sample selection process was as follows: First, each school was stratified by grade level (10th and 11th grades). Then, within each grade, the research team consulted with the physical education coordinators and homeroom teachers at each school to select 2–3 administrative classes based on class schedules and accessibility, ensuring that the selected classes could accommodate the questionnaire distribution schedule. All students in the selected classes were invited to participate in the survey; no random sampling was conducted at the individual level. In accordance with Schoemann’s criteria, the minimum sample size was calculated using an R program, with a statistical power of 0.80, 20,000 Monte Carlo simulations per iteration, and a 95% confidence level; the minimum sample size was calculated to be 197 cases (18). The questionnaires will be distributed in person, with the research team liaising with the heads of physical education and form tutors at the four secondary schools to agree on the timing, year groups, classes and number of students involved. In June 2023, 1,050 questionnaires were distributed on-site. Three of the schools distributed and collected the questionnaires during evening study periods, whilst another school conducted the survey during the midday break in classrooms. Before the participants completed the questionnaires, detailed instructions were provided regarding the points to note when filling them in (including the various question types and requirements), and any queries raised by the participants were addressed promptly during the process. The research team used Microsoft Excel 2016 to enter data from the returned paper questionnaires, screening and excluding questionnaires where “more than 5% of questions were missing”, “answers did not match the item”, or “selected ‘special circumstances’ (fractures, sprains, menstruation, sports competitions, etc.) for PAQ-CN Question 9”. A total of 891 questionnaires were ultimately collected, representing a response rate of 84.9%; of these, 841 were valid, yielding a validity rate of 94.4%. Among the respondents, 535 were male (63.6%) and 306 were female (36.4%); 318 were in Year 10 (37.8%) and 523 were in Year 11 (62.2%) (Table 1).

**Table 1:** Demographic distribution of the survey respondents (*n* = 841).

Variable	Category	Number of people	Percentage (%)
Gender	Male	535	63.6
	Female	306	36.4
Grade	10th grade	318	37.8
	11th grade	523	62.2

### 2.2 Research tools

#### 2.2.1 Physical activity questionnaire for adolescents

The physical activity questionnaire developed by Guo Qiang et al. for adolescents was used. This is a retrospective self-administered questionnaire, which asks participants to report their physical activity behavior over the past 7 days. It comprises 10 questions covering the frequency, type and duration of physical activity, and measures the final score of adolescents’ physical activity levels using a predefined scoring system (19). The PAQ-CN is scored using a standardised scoring method to produce a continuous score ranging from 1 to 5, with higher scores indicating a higher level of physical activity. To facilitate the description of sample characteristics, this study categorized scores into three levels—low (≤2), moderate (2 < PAQ-CN score ≤ 3) and high (>3)—using the cut-off values from existing studies; however, in subsequent regression analyses, the raw continuous scores were used as the dependent variable to maximise the retention of data variability.

#### 2.2.2 Physical activity cognition questionnaire for adolescents

The PACO questionnaire primarily covers two dimensions: the impact of physical activity on academic performance and physical health. It comprises a total of six questions, with each item designed using a five-point Likert scale, scored in descending order as 5, 4, 3, 2, and 1. The scores are then totalled and the mean is calculated (20). In this study, the results of the reliability analysis indicated that Cronbach’s *α* was 0.735, reflecting good internal consistency reliability and confirming the questionnaire’s suitability for investigating secondary school students’ perceptions of physical activity.

#### 2.2.3 Adolescent social support for physical activity scale

Using the ASAFA developed by de Farias Júnior JC, Mendonça G (21), this study investigated the levels of parental and peer support for adolescents’ physical activity. The final revised version of the scale is not limited by demographic or racial characteristics and is suitable for surveys on social support for adolescents’ physical activity across different regions. Both parental support for physical activity and peer support for physical activity consist of five items, with response options of “Never,” “Rarely,” “Often,” and “Always,” corresponding to scores of 1, 2, 3, and 4, respectively. The final score is calculated by summing the scores for each item; a higher score indicates a higher level of support. Peer support is categorized into two levels: high (14.0 ≤ score ≤ 20.0) and low (5.0 ≤ score < 14.0); parental support is similarly categorized into two levels: high (12.0 ≤ score ≤ 20.0) and low (5.0 ≤ score < 12.0). In this study, the Cronbach’s alpha for the scale was 0.897, indicating that the scale has good reliability.

### 2.3 Data analysis

**Figure 1:**
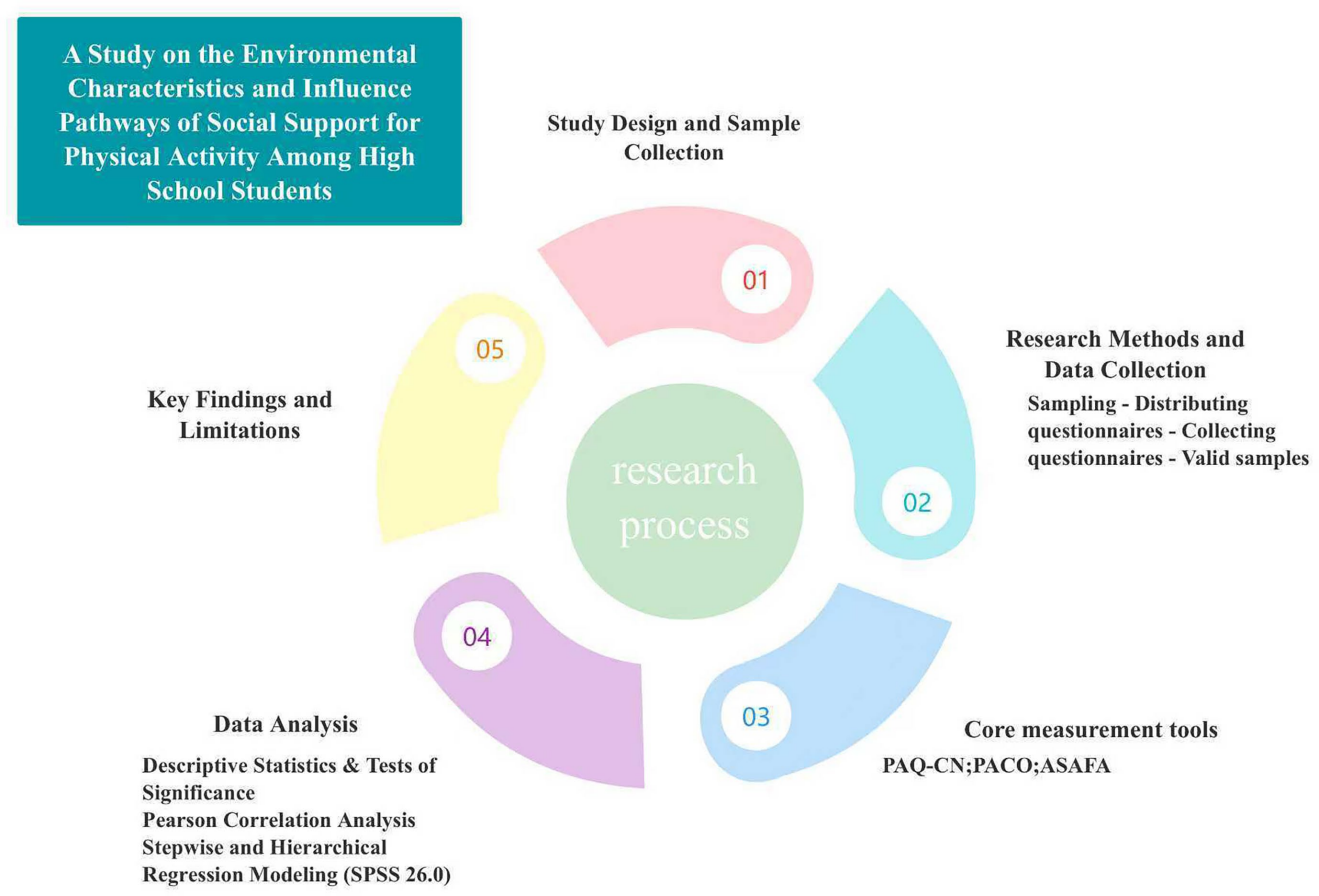
Research design process.

This study utilized IBM SPSS Statistics 26.0 to perform statistical analyses on the various variables, with the raw continuous scores for physical activity levels among secondary school students (PAQ-CN scores) serving as the dependent variable, and peer support, parental support and perceptions of physical activity as independent variables; gender, year group and school were included in the analysis as control variables. Firstly, frequency analysis and descriptive statistics will be used to examine the basic distribution characteristics of the sample, as well as the means and standard deviations of each variable; an independent *t*-test will then be employed to analyse differences in physical activity levels, peer support and parental support across different genders and year groups. Secondly, Pearson’s correlation analysis was used to examine the bivariate relationships between physical activity levels and peer support, parental support, and perceptions of physical activity, thereby laying the groundwork for subsequent regression analysis. Subsequently, to initially explore the contribution of variables and identify predictors, a stepwise regression method was employed, with a inclusion criterion of an *F*-statistic probability ≤ 0.05 and an exclusion criterion of an *F*-statistic probability ≥ 0.10. Given that stepwise regression carries a risk of overfitting and that results depend on sample characteristics, this study primarily utilized a theory-driven hierarchical multiple regression model. After controlling for gender, year group and school, the model progressively incorporated perceptions of physical activity, parental support and peer support to examine the independent explanatory power of each variable on physical activity levels, whilst reporting changes in model fit (Δ*R*^2^) and multicollinearity diagnostics (VIF) (Figure 1).

## 3 Results

### 3.1 Descriptive statistics and analysis of differences in physical activity levels among high school students in relation to peer support and parental support

According to the PAQ-CN assessment criteria, a higher physical activity level score indicates a higher level of daily physical activity. The findings of this study show that, in terms of the distribution of physical activity levels (based on cutoff values), 63.4% of high school girls were classified as having a low level of physical activity, which is higher than the corresponding proportion of 36.1% among boys; at the same time, only 6.9% of girls were classified as having a high level of physical activity, which is significantly lower than the 24.5% among boys. By grade level, 42.8% of 10th graders and 48.0% of 11th graders were classified as having low levels of physical activity, with the proportion slightly higher among 11th graders (Table 2). An independent samples *t*-test was conducted using the raw continuous total score of the PAQ-CN as the analysis variable. The results indicated that the total physical activity score for male students (2.39 ± 0.74) was significantly higher than that for female students (1.93 ± 0.59) (*t* = 9.258, *p* < 0.001), but there was no statistically significant difference between grades (*p* > 0.05) with scores for first-year (2.22 ± 0.73) and second-year (2.21 ± 0.73) students being similar (Table 3).

**Table 2:** Descriptive statistics on physical activity levels and social support levels among secondary school students.

Variable	Category	Male (*n* = 535)	Female (*n* = 306)	10th grade (*n* = 318)	11th grade (*n* = 523)
Physical activity	low level	193 (36.1%)	194 (63.4%)	136 (42.8%)	251 (48.0%)
	Intermediate level	211 (39.4%)	91 (29.7%)	118 (37.1%)	184 (35.2%)
	High-level	131 (24.5%)	21 (6.9%)	64 (20.1%)	88 (16.8%)
Parental support	Low support	238 (44.5%)	132 (43.1%)	132 (41.5%)	238 (45.5%)
	High support	297 (55.5%)	174 (56.9%)	186 (58.5%)	285 (54.5%)
Peer support	Low support	193 (36.1%)	175 (57.2%)	133 (41.8%)	235 (44.9%)
	High support	342 (63.9%)	131 (42.8%)	185 (58.2%)	288 (55.1%)

The classifications of “low/medium/high” and “high/low” support levels mentioned above are used solely to describe the frequency distribution characteristics of the sample, in order to present the basic situation of each subgroup.

**Table 3:** An analysis of variations in physical activity levels and social support among secondary school students.

Variable	Male	Female	*t*	*p*	10th grade	11th grade	*t*	*p*
Physical activity (total score)	2.39 ± 0.74	1.93 ± 0.59	9.258	<0.001***	2.22 ± 0.73	2.21 ± 0.73	0.196	0.846
Parental support (total score)	12.28 ± 3.58	11.94 ± 3.71	1.289	0.098	12.31 ± 3.53	12.06 ± 3.69	0.957	0.339
Peer support (total score)	14.46 ± 4.18	12.55 ± 3.89	6.623	<0.001***	13.75 ± 4.16	13.78 ± 4.20	−0.100	0.920

* indicates *p* < 0.05, ** indicates *p* < 0.01.

In terms of social support, there was a significant gender difference in the total score for peer support (*t* = 6.623, *p* < 0.001), with boys scoring higher (14.46 ± 4.18) than girls (12.55 ± 3.89); In terms of distribution by score level, 63.9% of male students received high levels of peer support (≥14 points), compared to only 42.8% of female students, further reflecting the gender differences in peer support. Furthermore, there was no significant difference in the total parental support score by gender (*p* > 0.05); the scores for boys (12.28 ± 3.58) were similar to those for girls (11.94 ± 3.71), and the proportions of students reporting high levels of support were also similar between the two groups, at 55.5 and 56.9%, respectively. Across grade levels, there were no significant differences in total scores for peer support and parental support between first-year high school students (13.75 ± 4.16/12.31 ± 3.53) and second-year high school students (13.78 ± 4.20/12.06 ± 3.69) (*p* > 0.05) (Table 3).

### 3.2 A correlation analysis of physical activity among high school students and its determinants

In this study, Pearson’s correlation analysis was conducted on physical activity levels, perceptions of physical activity, parental support and peer support. The results show (Table 4) that physical activity was significantly positively correlated with parental support (*r* = 0.275), peer support (*r* = 0.495) and perceptions of physical activity (*r* = 0.303) (*p* < 0.01); Parental support was significantly positively correlated with peer support (*r* = 0.488) and perceptions of physical activity (*r* = 0.265) (*p* < 0.01); peer support was also significantly positively correlated with perceptions of physical activity (*r* = 0.352, *p* < 0.01).

**Table 4:** Correlation analysis of physical activity and individual factors in high school students.

Categories	Physical activity	Parental support	Peer support	Perceptions of physical activity
Physical activity	1			
Parental support	0.275**	1		
Peer support	0.495**	0.488**	1	
Perceptions of physical activity	0.303**	0.265**	0.352**	1
M	2.22	12.16	13.77	4.28
SD	0.79	3.63	4.18	0.72

**: Significant at the 0.01 level (two-tailed); M: mean; SD: standard deviation.

### 3.3 Path analysis of a regression model for factors influencing physical activity among high school students

#### 3.3.1 Stepwise regression model of factors influencing physical activity

A regression model was constructed using stepwise regression, with the level of physical activity among secondary school students (raw continuous score on the PAQ-CN) as the dependent variable, and perceptions of physical activity, parental support and peer support as independent variables (Table 5). In Model 1, with peer support included, the adjusted *R*^2^ was 0.244, Δ*R*^2^ was 0.245, and the standardised regression coefficient *β* was 0.495. This indicates that peer support accounts for 24.4% of the variation in physical activity and that peer support is a significant predictor of physical activity levels (*F* = 272.740, *p* < 0.001). Model 2 incorporated perceptions of physical activity on top of Model 1, resulting in an increase in adjusted *R*^2^ to 0.262; that is, this variable accounts for 26.2% of the variation in physical activity. Furthermore, both peer support (*β* = 0.444) and perceptions of physical activity (*β* = −0.146) were found to be significant positive predictors of physical activity levels (*F* = 150.353, *p* < 0.001), with peer support making a relatively greater contribution; parental support, however, was excluded during the stepwise regression process as it did not meet the inclusion criteria and therefore did not demonstrate a contribution to physical activity levels.

**Table 5:** Stepwise regression analysis of physical activity and physical activity cognition, parental support, and peer support among high school students.

Categories	Model	Variable	*β*	95%CI	*p*	*F*	VIF	Adjusted *R*^2^	Δ*R*^2^
Overall	1	Peer support	0.495	[0.415, 0.526]	<0.001	272.740***	1.000	0.244	0.245
	2	Peer support	0.444	[0.362, 0.481]	<0.001	150.353***	1.142	0.262	0.019
		PACO	−0.146	[−0.228, −0.092]	<0.001		1.142		
Male	1	Peer support	0.500	[0.409, 0.550]	<0.001	177.865***	1.000	0.249	0.250
	2	Peer support	0.445	[0.352, 0.503]	<0.001	97.503***	1.166	0.265	0.018
		PACO	−0.145	[−0.243, −0.072]	<0.001		1.166		
	3	Peer support	0.395	[0.294, 0.464]	<0.001	67.490***	1.492	0.272	0.008
		PACO	−0.137	[−0.234, −0.063]	<0.001		1.175		
		Parental support	0.104	[0.020, 0.205]	0.017		1.375		
Female	1	Peer support	0.383	[0.247, 0.432]	<0.001	52.221***	1.000	0.144	0.147

The dependent variable is physical activity (raw continuous score on the PAQ-CN); ***indicates *p* < 0.001; constant terms are omitted; the model analysis controlled for the variable ‘year group’.

To further explore gender differences in the factors associated with physical activity among secondary school students, stepwise regression analyses were conducted separately for male and female samples. For male students, the explanatory power of the variables included in each model and the standardised regression coefficients were generally similar to those found in the overall sample. It is worth noting that parental support was included in the model of factors influencing physical activity among male students (*β* = 0.104, *p* < 0.05), indicating that it significantly and positively predicts their level of physical activity. In the sample of girls, only peer support was included in the regression model, with an adjusted *R*^2^ of 0.144 and a Δ*R*^2^ of 0.147, indicating that it has a significant positive predictive effect on girls’ levels of physical activity (*β* = 0.383, *F* = 52.221, *p* < 0.001); whereas parental support and perceptions of physical activity were not included in the final model.

#### 3.3.2 Hierarchical regression model of factors influencing physical activity

Given that gender, year group and school may confound the model results, this study included them as control variables in a hierarchical regression model to examine more accurately the independent contributions of each influencing factor to physical activity levels. As shown in Table 6, Model 1 included only control variables, with ΔR^2^ of 0.096 and an adjusted R^2^ of 0.093. These results indicate that gender and school account for 9.3% of the variance in physical activity levels, and the model as a whole reached statistical significance (*F* = 29.771, *p* < 0.001). Model 2 further included three independent variables—perceptions of physical activity, parental support and peer support—in addition to the control variables. Compared with Model 1, Model 2 showed a significant improvement in explanatory power, with an adjusted *R*^2^ of 0.295 and a Δ*R*^2^ of 0.300 (*F* = 59.470, *p* < 0.001). After controlling for the confounding effects of gender and school, perceptions of physical activity (*β* = −0.121, *p* < 0.001) and peer support (*β* = 0.380, *p* < 0.001) continued to exert a significant positive predictive effect on physical activity levels, whilst the regression coefficient for parental support did not reach statistical significance (*p* > 0.05) (Table 6).

**Table 6:** Hierarchical regression analysis of high school students’ physical activity and individual variables.

Model	Variable	*β*	95%CI	*p*	*F*	Δ*R*^2^	VIF	Adjusted *R*^2^
1	Constant		[2.925, 3.424]	<0.001	29.771***	0.096		0.093
	Gender	−0.267	[−0.550, −0.332]	<0.001			1.052	
	Grade	−0.057	[−0.203, 0.015]	0.091			1.070	
	School	−0.115	[−0.124, −0.033]	<0.001			1.080	
2	Variable		[1.577, 2.256]	<0.001	59.470***	0.300		0.295
	Constant	−0.169	[−0.378, −0.179]	<0.001			1.130	
	Gender	−0.037	[−0.157, 0.036]	0.221			1.078	
	Grade	−0.071	[−0.089, −0.008]	0.019			1.090	
	Parental support	0.052	[−0.016, 0.129]	0.124			1.358	
	Peer support	0.380	[0.295, 0.427]	<0.001			1.495	
	PACO	−0.121	[−0.020, −0.065]	<0.001			1.176	

The dependent variable is physical activity (raw continuous score on the PAQ-CN); ***indicates *p* < 0.001.

Table 6 Hierarchical regression analysis of high school students’ physical activity and individual variables.

## 4 Discussion

### 4.1 Distribution characteristics of physical activity levels and their determinants

The level of physical activity among adolescents is closely linked to factors in their social environment. This study examines the distribution characteristics of social support environments and their association with the level of physical activity among secondary school students from the perspective of interpersonal relationships within school and family settings, thereby providing a theoretical framework for promoting social support for physical activity among secondary school students. High school students are at a critical stage of transition from adolescence to adulthood, and this is also a period marked by significant academic pressure. Previous research has shown that physical activity can enhance academic performance among secondary school students, alleviate academic stress (22), and protect mental health (23), while also being closely associated with physical health, life satisfaction, and subjective exercise experiences (24). However, recent surveys indicate that only about 30% of Chinese children and adolescents meet the recommended levels of physical activity, with boys exceeding girls and middle school students falling short of elementary school students (25). This study focuses on high school students, who represent a vital human resource for the nation’s future development; their physical health is crucial to the nation’s sustainable development. Therefore, exploring the social support mechanisms for physical activity among this group holds significant practical importance.

This study found that the overall level of physical activity among high school students is low. The survey results indicate that male high school students engage in significantly more physical activity than female students, although there were no significant differences across grade levels. 63.4% of female high school students were classified as having low levels of physical activity, which is significantly higher than the 36.1% of male students; meanwhile, only 6.9% of female students were classified as having high levels of physical activity, a proportion that is notably lower than the 24.5% of male students. These findings are consistent with those of numerous other studies (26). This indicates that girls generally engage in physical activity at lower levels during adolescence and that a “polarization” phenomenon exists (only a small number of girls have high levels of physical activity, while the majority are physically inactive). According to the socio-ecological model, individual behavior is influenced by the combined effects of micro-, meso-, and macro-level systems. The physical activity levels of adolescent girls are shaped by the interaction of macro-level sociocultural factors (such as gender stereotypes), meso-level environmental factors (such as school physical education resources and peer influence), and micro-level individual factors. As a result, most girls remain physically inactive due to a lack of support systems, while a small number of girls who have positive family or peer support can maintain high levels of physical activity. At the grade level, there were no significant differences in physical activity levels between freshmen and sophomores; however, in terms of scores, the proportion of sophomores with low activity levels was slightly higher than that of freshmen, which may be related to increased academic pressure and reduced free time (27).

Regarding social support, this study found that boys received significantly more peer support than girls, and a higher proportion of boys reported receiving high levels of support. This finding is consistent with the results of studies by Lawler (28) and Clare (29), indicating that high school boys in this sample have an advantage in accessing peer support. When it comes to socializing and sports, boys tend to form and maintain friendships through team sports such as basketball and soccer, while girls engage in more reserved social interactions during sports, resulting in a lower sense of support (30). Neither gender nor grade level showed significant differences in parental support, indicating that parental support for children’s physical activity is relatively stable and is rarely adjusted based on changes in the child’s age or gender. However, studies by Hu (31) and Yang (32) found that parental participation in sports has a positive impact on adolescents’ moderate-to-vigorous physical activity and their motivation to engage in such activities; however, these studies did not distinguish between educational levels. This study suggests that among high school students, the role of parental support may diminish compared to childhood, while the influence of peer support gradually increases. This aligns with the typical patterns of adolescent development, characterized by growing autonomy and the increasing importance of peer relationships (33).

Peer relationships play a significant role in adolescents’ acquisition of motor skills, participation in physical activity, and psychological development. Existing research indicates that peer support, family support, and individual-level self-efficacy collectively influence physical activity (34). Support from teachers, parents, and peers provides adolescents with a sense of self-efficacy, which can stimulate their intrinsic motivation and thereby influence their physical activity behavior. Students experience greater stress relief when engaging in group physical activities, and harmonious interpersonal relationships among female students have a positive impact on their physical activity (35). However, in this study, both the level of physical activity and the amount of social support received by female students were relatively low. These findings highlight the complexity of gender differences in physical health promotion and suggest that intervention measures need to be more targeted.

### 4.2 The impact of social support on physical activity levels

The results of this study indicate that peer support, parental support and perceptions of physical activity are all significantly positively correlated with levels of physical activity, with peer support showing the strongest correlation. Further analysis revealed that, after controlling for gender, year group and school variables, peer support and perceptions of physical activity continued to have a significant positive predictive effect on levels of physical activity, whereas the independent contribution of parental support was not statistically significant. This suggests that, among secondary school students, peer support and individual perceptions are more direct contributing factors. According to social support theory, social support—in the form of emotional, informational, and instrumental support—can alleviate negative emotions individuals experience when facing stress, thereby increasing their engagement in healthy behaviors (36). For high school students, peer support in daily life and academic settings is characterized by its immediacy, continuity, and emotional connection; it can directly promote physical activity through various means, such as shared participation and mutual encouragement. Hamilton’s research also confirms that adolescents can gain better physical and emotional experiences through shared participation within positive peer relationships (37). In contrast, even when parental support is present, it often takes the form of “external provision” rather than “internal integration.” Furthermore, given that high school students spend long hours at school and have limited interaction with their parents, this support is difficult to directly translate into motivation and behavior related to physical activity.

The results of the gender-stratified analysis further reveal differences in the association pathways: physical activity among boys was associated with peer support, perceptions of physical activity, and parental support, whereas only peer support was included in the regression model for girls. These findings suggest that the mechanisms underlying physical activity among boys are relatively multifaceted, whereas girls demonstrate a singular reliance on peer support. It is evident, therefore, that the relationship between social support and physical activity is not universally uniform; the extent of its effects and the pathways through which it operates may vary depending on individual characteristics. In this study, the factors associated with physical activity among girls exhibited a singular nature, with their physical activity being more dependent on the availability and quality of peer support, whilst showing relatively lower sensitivity to other types of support. From a gender-based theoretical perspective, during the socialization process, women are assigned greater social expectations regarding emotional expression and relationship maintenance, and the construction of their interpersonal relationships places greater emphasis on intimacy, emotional sharing, and mutual support (38). Consequently, in physical activity settings, they have higher expectations regarding the reliability, emotional investment, and responsiveness of the support they receive from peers, and they also have stricter standards for the quality of that support. Furthermore, perceptions of physical activity were not included in the regression model for the female sample, indicating that cognitive factors have limited explanatory power regarding their actual physical activity behavior. According to social cognitive theory, individual behavior is the result of the interaction among the individual, the environment, and behavioral factors (39). For female students, even when they possess positive attitudes toward physical activity, their actual decision-making processes may still be constrained by factors such as social anxiety, body image concerns, and self-efficacy regarding physical ability; these factors may weaken the direct influence of cognitive factors on behavior. Therefore, future research could further explore the mediating mechanisms and moderating variables involved in the translation of physical activity attitudes into behavior among female students, to elucidate the complex pathways underlying their behavioral decisions more comprehensively.

Compared with previous studies, this research not only confirms the role of peer support in promoting physical activity among adolescents but also reveals the contextual factors influencing this effect through a gender-stratified analysis. Previous research has largely treated social support as a single, unifying factor (40), with little attention paid to gender differences in its mechanisms of action. By introducing gender as a variable, this study has deepened our understanding of the relationship between social support and physical activity, thereby providing a basis for the development of tailored intervention strategies. Furthermore, differences at the school level are significantly associated with physical activity. The four provincial key high schools examined in this study each have distinct educational philosophies and values, which may be indirectly reflected in the practical implementation of physical education curricula, physical activities and sporting events, thereby influencing students’ participation in physical activity. From the perspective of the socio-ecological model, individual behavior is the result of the interaction of factors at the micro, meso, and macro levels (41). The differences in practices regarding physical education curricula, physical activities, and sports events observed in the four provincial key high schools surveyed in this study influence students’ opportunities for physical activity, potentially either promoting or limiting their participation. Future research could further explore how specific school-level characteristics interact with individual and interpersonal factors to jointly shape high school students’ physical activity patterns.

## 5 Conclusion

This study found that (1) physical activity levels among high school students were generally low, with significant gender differences; specifically, male high school students had significantly higher physical activity levels than female students, while no significant difference was observed between first- and second-year students. Regarding social support, male students received higher levels of peer support and parental support than female students, with peer support being higher than parental support. Furthermore, peer support had a greater predictive effect on physical activity levels than parental support, suggesting that peers have become a more influential source of support for adolescents during high school. (2) The pathways influencing physical activity among secondary school students show gender differences: physical activity among boys is associated with peer support, parental support and perceptions of physical activity, whereas physical activity among girls is significantly associated only with peer support. These findings reveal gender differences in the role of social support and provide empirical evidence for developing gender-specific intervention strategies.

To further promote the physical and mental well-being of high school students, this study offers the following recommendations based on its findings: (1) Schools should create a rich and diverse range of physical activity opportunities for high school students, paying particular attention to the needs of female students. By designing physical activities that align with their interests and emphasize cooperation and social interaction, schools can stimulate their participation in physical activity. (2) Schools should strengthen peer interaction and communication, leveraging the central role of peer support in physical activity participation to enhance the sustainability and retention of physical activity behaviors. (3) The provision of social support must align with the patterns of students’ physical and mental development and offer high-quality, diverse forms of support to promote a comprehensive increase in physical activity among high school students.

However, this study does have certain limitations: firstly, with regard to the sampling method, this study employed non-probability convenience sampling, with the sample drawn solely from four provincial key high schools; furthermore, classes were selected based on accessibility rather than random selection. Although the sample size met statistical requirements, the representativeness of the sample is limited. Future research could adopt probability sampling or multi-centre large-sample sampling to enhance the generalisability of the findings. Secondly, this study employed a cross-sectional design, with data collection taking place at a single point in time; consequently, all analytical findings can only reveal associations between variables. Future research should adopt longitudinal or experimental intervention designs to further investigate the causal relationships and dynamic evolution between variables. Thirdly, the core variables in this study were collected via self-report questionnaires, which may be subject to recall bias and social desirability. It is recommended that future studies incorporate objective measurement tools, such as accelerometers, to assess physical activity levels, thereby enhancing the objectivity of the data. Fourthly, with regard to statistical methods, stepwise regression analysis carries a risk of model instability and overfitting due to the nature of its algorithm; therefore, caution should be exercised when generalizing its results. It is recommended that future research adopt structural equation modelling to enhance the generalisability of the findings.

## Data Availability

The original contributions presented in the study are included in the article/supplementary material, further inquiries can be directed to the corresponding author.

## References

[1] Bull FC Al-Ansari SS Biddle S Borodulin K Buman MP Cardon G . World Health Organization 2020 guidelines on physical activity and sedentary behaviour. Br J Sports Med. (2020) 54:1451–62. doi: 10.1136/bjsports-2020-102955 33239350 PMC7719906

[2] Shilton T Milton K. Achieving advocacy success-the international society for physical activity and health's long-term strategy to advance physical activity as a priority in global health policy. J Phys Act Health. (2024) 21:1446–52. doi: 10.1123/jpah.2024-0214, 39222920

[3] Chaput JP Willumsen J Bull F Chou R Ekelund U Firth J . WHO guidelines on physical activity and sedentary behaviour for children and adolescents aged 5-17 years: summary of the evidence. Int J Behav Nutr Phys Act. (2020) 17:141. doi: 10.1186/s12966-020-01037-z 33239009 PMC7691077

[4] Council TCCotCPoCatS. The “healthy China 2030” planning outline. (2016). Available online at: https://www.gov.cn/zhengce/2016-10/25/content_5124174.htm2016.

[5] Xielin Z Mu Z Lu C Bo L. Cross-lagged analysis of mobile phone addiction, bedtime procrastination, and exercise self-efficacy among university students. J. Exerc. Sci. Fit. (2025) 23:369–76. doi: 10.1016/j.jesf.2025.07.003, 40703407 PMC12284518

[6] Qin R Wu JT Zang WL Zhang D Feng XW. Prevalence of insufficient daily physical activity and its association with health indicators among Chinese primary and secondary school students: a cross-sectional study. Front Public Health. (2026) 14. doi: 10.3389/fpubh.2026.1732510, 41717627 PMC12913497

[7] Net G. The overall rate of myopia among children and adolescents in China is 52.7%. (2023). Available online at: https://baijiahao.baidu.com/s?id=1776794020058869131&wfr=spider&for=pc

[8] China TWCotRoCHDi. Key points interpretation of "China cardiovascular health and disease report 2022". Chin J Cardiovasc Med. (2023) 28:297–312.

[9] Lieber SB Moxley J Mandl LA Reid MC Czaja SJ. Social support and physical activity: does general health matter? Eur Rev Aging Phys Act. (2024) 21:16. doi: 10.1186/s11556-024-00347-6 38902616 PMC11188280

[10] Eagle DE Hybels CF Proeschold-Bell RJ. Perceived social support, received social support, and depression among clergy. J Soc Pers Relat. (2019) 36:2055–73. doi: 10.1177/0265407518776134

[11] Yücekaya MA Ugras S Sagin AE Çetin A Iconomescu TM Talaghir LG. Relationships among social support, healthy lifestyle beliefs, physical literacy, and enjoyment of physical activity: a moderated mediation model. Front Public Health. (2025) 13. doi: 10.3389/fpubh.2025.1617124, 40832035 PMC12360299

[12] Prado CV Lima AV Fermino RC Añez CRR Reis RS. Social support and physical activity in adolescents from public schools: the importance of family and friends. Cad Saude Publica. (2014) 30:827–38. doi: 10.1590/0102-311X00014313, 24896057

[13] Kim J Kim J Kim Y Han A Nguyen MC. The contribution of physical and social activity participation to social support and happiness among people with physical disabilities. Disabil Health J. (2021) 14:100974. doi: 10.1016/j.dhjo.2020.100974, 32811784

[14] Hamilton K White KM. Parental physical activity: exploring the role of social support. Am J Health Behav. (2010) 34:573–84. 20524887 10.5993/ajhb.34.5.7

[15] Lin H Chen H Liu Q Xu J Li S. A meta-analysis of the relationship between social support and physical activity in adolescents: the mediating role of self-efficacy. Front Psychol. (2024) 14. doi: 10.3389/fpsyg.2023.1305425, 38282843 PMC10811609

[16] Lee Y Park S. Understanding of physical activity in social ecological perspective: application of multilevel model. Front Psychol. (2021) 12. doi: 10.3389/fpsyg.2021.622929, 33746840 PMC7973361

[17] Liang F Yu H Li F Li X. Relationship between parental physical activity and adolescents’ exercise cognition: the mediating role of family activity support. Front Public Health. (2025) 13. doi: 10.3389/fpubh.2025.1685991, 41409694 PMC12705581

[18] Schoemann AM Boulton AJ Short SD. Determining power and sample size for simple and complex mediation models. Soc Psychol Personal Sci. (2017) 8:379–86. doi: 10.1177/1948550617715068

[19] Guo Qiang WX Jiang Jianbao. Research on physical activity and sedentary behavior patterns of children and adolescents in China. Sports Sci. (2017) 37:17–29. doi: 10.16469/j.css.201707003

[20] Coil YI Welk GJ Beyler NK Silva P Heelan KA. Estimating minutes of physical activity from the physical activity questionnaire for adolescents (PAQ-A). Med Sci Sports Exerc. (2010) 42:525–6. doi: 10.1249/01.MSS.0000385289.25830.1e

[21] LAD JJC Reis RS . Development and validation of a questionnaire measuring factors associated with physical activity in adolescents. Rev Bras Saude Mater Infant. (2011) 11:301–12.

[22] Che L Liu D Tie C. Physical activity and academic performance in adolescents: chain mediation through self-regulation and self-efficacy with gender and urban–rural differences. Front Educ. (2026) 10. doi: 10.3389/feduc.2025.1686270

[23] Molcho M Gavin A Goodwin D. Levels of physical activity and mental health in adolescents in Ireland. Int J Environ Res Public Health. (2021) 18. doi: 10.3390/ijerph18041713, 33578906 PMC7916674

[24] Chmelík F Frömel K Groffik D Mitás J. Physical activity and life satisfaction among adolescents before and during the COVID-19 pandemic. Acta Psychol. (2023) 241:104081. doi: 10.1016/j.actpsy.2023.104081, 37976920

[25] Fan X Cao ZB. Physical activity among Chinese school-aged children: national prevalence estimates from the 2016 physical activity and Fitness in China-the youth study. J Sport Health Sci. (2017) 6:388–94. doi: 10.1016/j.jshs.2017.09.006, 30356592 PMC6189233

[26] Arumi-Prat I Cirera-Viñolas E McKenna J Puig-Ribera A. Gender differences in barriers to sports participation on the transition from adolescence to young adulthood in a mediterranean region. Prev Med Rep. (2025) 58:103226. doi: 10.1016/j.pmedr.2025.103226, 40918685 PMC12410169

[27] He H Yang Y Sun J Wang F Zhang W Zhu F. Effects of school-based physical activity on academic achievement in children and adolescents: a systematic review and meta-analysis. Front Public Health. (2025) 13. doi: 10.3389/fpubh.2025.1651883, 41036117 PMC12479424

[28] Lawler M Heary C Shorter G Nixon E. Peer and parental processes predict distinct patterns of physical activity participation among adolescent girls and boys. Int J Sport Exerc Psychol. (2022) 20:497–514. doi: 10.1080/1612197X.2021.1891118

[29] Clare M Lenhart AH Kang Y Daly BP Michael D. Brown, Freda Patterson. Gender disparity in structured physical activity and overall activity level in adolescence: evaluation of youth risk behavior surveillance data. Int Sch Res Notices. (2012):674936. doi: 10.5402/2012/674936

[30] Bang H Chang MD Kim S. Team and individual sport participation, school belonging, and gender differences in adolescent depression. Child Youth Serv Rev. (2024) 159. doi: 10.1016/j.childyouth.2024.107517

[31] Hu YTY Zhang J . Research on the impact of parental factors on adolescents' engagement in high-intensity physical activities. Chin Sports Sci. (2017) 53:14–21. doi: 10.16470/j.csst.201703003

[32] Yang J Peng XC Liu X. The perspective of holistic theory: the pathway of parental support in promoting physical activity among adolescents. Sports Sci. (2019) 40:115–20. doi: 10.13598/j.issn1004-4590.2019.02.014

[33] Henson J Yates T Biddle SJH Edwardson CL Khunti K Wilmot EG . Associations of objectively measured sedentary behaviour and physical activity with markers of cardiometabolic health. Diabetologia. (2013) 56:1012–20. doi: 10.1007/s00125-013-2845-9, 23456209

[34] Morrissey JL Janz KF Letuchy EM Francis SL Levy SM. The effect of family and friend support on physical activity through adolescence: a longitudinal study. Int J Behav Nutr Phys Act. (2015) 12:103. doi: 10.1186/s12966-015-0265-6, 26289232 PMC4545918

[35] Pluhar E McCracken C Griffith KL Christino MA Sugimoto D Meehan WP. Team sport athletes may be less likely to suffer anxiety or depression than individual sport athletes. J Sports Sci Med. (2019) 18:490–6. 31427871 PMC6683619

[36] Lei XQ Wu H Deng ZH Ye Q. Self-disclosure, social support and postpartum depressive mood in online social networks: a social penetration theory perspective. Inf Technol People. (2023) 36:433–53. doi: 10.1108/ITP-12-2020-0825

[37] Hamilton K Warner LM Schwarzer R. The role of self-efficacy and friend support on adolescent vigorous physical activity. Health Educ Behav. (2017) 44:175–81. doi: 10.1177/1090198116648266, 27226431

[38] Cai FY Wang YJ Jin SY. The impact of social media addiction on college students' mental health through social support and resilience. Sci Rep. (2026) 16:5087. doi: 10.1038/s41598-026-35779-w, 41530270 PMC12877163

[39] Balla J Polet J Kokko S Hirvensalo M Vasankari T Lintunen T . Predicting adolescents' physical activity intentions: testing an integrated social cognition model. Int J Behav Med. (2024) 31:41–54. doi: 10.1007/s12529-023-10156-3, 36949326 PMC10032623

[40] Oliveira AJ Lopes CS Rostila M Werneck GL Griep RH de Leon A . Gender differences in social support and leisure-time physical activity. Rev Saude Publica. (2014) 48:602–12. doi: 10.1590/S0034-8910.2014048005183, 25210819 PMC4181105

[41] Huang MR Sun HC Chen H Zhang YP Adams K Gao Z. Validation of physical activity correlates questionnaire from social ecological model in college students. J Clin Med. (2023) 12. doi: 10.3390/jcm12030777, 36769426 PMC9917748

